# Surgical vs. Conservative Treatment of Distal Radius Fractures in the Elderly: A Systematic Review and Meta-Analysis

**DOI:** 10.7759/cureus.75879

**Published:** 2024-12-17

**Authors:** Abdulelah A Alanazi, Abdulkarim M Alsharari, Nawaf H Alrumaih, Aseel I Alsudays, Amer K Alanazi, Mohamed Alhilali, Fatemah Bo Shagea, Mohammed M Al-Rawaf, Faisal J Alsiwat

**Affiliations:** 1 Orthopedic Surgery, King Saud Medical City, Riyadh, SAU; 2 Physiotherapy and Health Rehabilitation, Jouf University, Sakaka, SAU; 3 College of Medicine, Majmaah University, Al Majma'ah, SAU; 4 Research, Sulaiman Al Rajhi University, Al Bukayriyah, SAU; 5 Orthopedic Surgery, Majmaah University, Al Majma'ah, SAU; 6 College of Medicine, Umm Al-Qura University, Al Qunfudhah, SAU; 7 General Practice, I.M. Sechenov First Moscow State Medical University, Moscow, RUS; 8 College of Dentistry, King Saud University, Riyadh, SAU

**Keywords:** conservative management, dash score, distal radius fractures, elderly patients, grip strength, meta-analysis, pain intensity, randomized controlled trials, surgical treatment, volar plate fixation

## Abstract

Elderly patients with distal radius fractures (DRFs) pose a significant medical challenge due to their high incidence and related healthcare costs. Both surgical methods like volar plate fixation and conservative approaches such as casting are common, yet their relative effectiveness remains unclear. This review and meta-analysis compare surgical and conservative treatments, focusing on wrist functionality, upper extremity performance, grip strength, and pain after one year. A literature search was conducted in PubMed, Cochrane Library, Scopus, and Web of Science up to November 6, 2024, to find randomized controlled trials (RCTs) for DRFs in patients aged 65 and older. Thirteen RCTs with 2400 participants were included. After one year, there was no significant difference in wrist function between groups (mean difference (MD)=-1.24; 95% confidence interval (CI) -2.61 to 0.13; p=0.78; I²=78%), but upper limb function favored surgery as measured by the Disabilities of the Arm, Shoulder, and Hand (DASH) score (MD=-2.32; 95% CI -3.66 to -0.98; p=0.0007; I²=83%). Surgical treatment significantly improved grip strength (MD=3.82; 95% CI 1.55 to 6.09; p=0.001) but resulted in higher pain levels (MD=2.73; 95% CI 1.16 to 4.31; p=0.0007). Results showed substantial heterogeneity and publication bias. Surgical treatment offers slight functional advantages in grip strength and DASH scores but is associated with higher pain; conservative treatment remains viable with minimal differences in long-term wrist function. Further high-quality studies are necessary to address heterogeneity and publication bias.

## Introduction and background

Distal radius fractures (DRFs) are among the most common injuries encountered in clinical practice, particularly in the elderly population [1,2]. These injuries make up a significant proportion of fractures treated in individuals aged 65 and above, largely due to the higher prevalence of osteoporosis and increased susceptibility to falls in this age group [3,4]. The management of DRFs remains a subject of debate, with treatment options ranging from conservative methods, such as closed reduction and cast immobilization, to surgical procedures like volar plate fixation and percutaneous pinning [5-7]. Both approaches aim to restore wrist function, alleviate pain, and improve the patient's overall quality of life. However, the optimal treatment strategy, especially for older patients, remains unclear, as outcomes are often influenced by patient-specific factors, fracture characteristics, and the surgeon's expertise [8].

The controversy over treating DRFs in elderly individuals arises from the specific challenges this demographic presents. Older patients typically have multiple health issues, diminished physical resilience, and lower activity requirements compared to younger counterparts, which can affect treatment strategies. Conservative management is often preferred due to its straightforward nature and lower risk of anesthesia and surgical complications. Nevertheless, operative approaches have gained popularity recently, thanks to improved techniques and implant technologies that potentially offer better anatomical restoration and quicker rehabilitation. Despite these advancements, the question persists: Are the possible advantages of surgery worth the inherent risks and expenses, particularly in an aging population? The subject is thoroughly explored in the guidelines set forth by the American Academy of Orthopaedic Surgeons (AAOS) [9]. However, these guidelines neither recommend nor advise against surgical procedures for older adults with DRFs, citing insufficient evidence to prove that surgical outcomes are superior to non-surgical treatments [6]. Interestingly, two recent systematic reviews that compared the effectiveness of volar locking plates to cast immobilization in elderly patients with DRFs produced conflicting results [10,11].

Research findings and comprehensive reviews have produced mixed results, with certain studies indicating better functional outcomes and fewer complications in surgical approaches, while others report no substantial differences between surgical and non-surgical treatments. Further complicating matters is the increasing focus on patient-centered care, which aims to align treatment strategies with both clinical evidence and individual patient preferences. This highlights the necessity of compiling and analyzing high-quality evidence to inform clinical decision-making in this field. This systematic review and meta-analysis are motivated by the ongoing ambiguity and changing evidence surrounding the treatment of DRFs in elderly patients. Considering the substantial impact on patient well-being and healthcare expenses associated with these injuries, a thorough assessment of surgical versus conservative management approaches is essential. By systematically examining and aggregating data from randomized controlled trials (RCTs), this study seeks to fill a knowledge gap, emphasizing functional outcomes, complication frequencies, and quality-of-life measures at the one-year mark. The primary goal is to offer healthcare providers evidence-based knowledge to inform personalized treatment strategies and enhance care for older adults with DRFs.

## Review

Methods

Literature Search

A comprehensive literature search was conducted across multiple databases, including PubMed, Cochrane Library, Scopus, and Web of Science, to identify RCTs evaluating the effectiveness of surgical versus conservative treatment for DRFs in elderly patients. The search was performed up to November 6, 2024. Relevant terms related to DRFs, surgical treatment, conservative management, and outcomes in elderly populations were used.

Study Selection

Only RCTs comparing surgical (volar plate fixation or K-wire) versus conservative treatment (e.g., casting or splinting) for DRFs in patients aged 65 years or older were included. Studies were eligible if they provided data on at least one relevant outcome measure (e.g., wrist function, range of motion, grip strength, or pain). Studies were excluded if they did not report age-specific data, were not RCTs, or did not provide sufficient outcome data for analysis.

Outcome Measures

The primary outcome measure for this meta-analysis was wrist function at one-year follow-up, measured using the Patient-Rated Wrist Evaluation (PRWE). Secondary outcomes included upper limb function, assessed using the Disabilities of the Arm, Shoulder, and Hand (DASH) score, grip strength, range of wrist motion (flexion, extension, pronation, supination), and pain intensity. All outcomes were assessed at one-year follow-up, and measurements were recorded using validated instruments such as dynamometers and goniometers.

Data Extraction

Two independent reviewers conducted data extraction from the included studies using a predefined data extraction form. Information was collected on study characteristics (e.g., author, year of publication, sample size, study design), patient demographics (e.g., age, sex), treatment groups, and outcomes for each measure at one-year follow-up. Discrepancies between reviewers were resolved through discussion or by consulting a third reviewer.

Data Analysis

Meta-analysis was conducted using RevMan Version 5.4 (The Cochrane Collaboration, London, England, United Kingdom). The mean difference (MD) with 95% confidence intervals (CI) was used to summarize the results for continuous outcomes. A random-effects model was applied to account for the expected variation between studies. Heterogeneity across studies was assessed using the I² statistic, where a value greater than 50% indicated significant heterogeneity. Publication bias for the primary outcome, wrist function, was assessed visually using a funnel plot.

Risk of Bias

The risk of bias in the included studies was assessed using the Cochrane Collaboration Risk of Bias Tool, which evaluates risk across several domains: selection bias, performance bias, detection bias, attrition bias, and reporting bias. Each study was categorized as having low, unclear, or high risk of bias in each domain. The overall risk of bias for each study was determined based on the combined risk across domains.

Results

Study Selection

**Figure 1:**
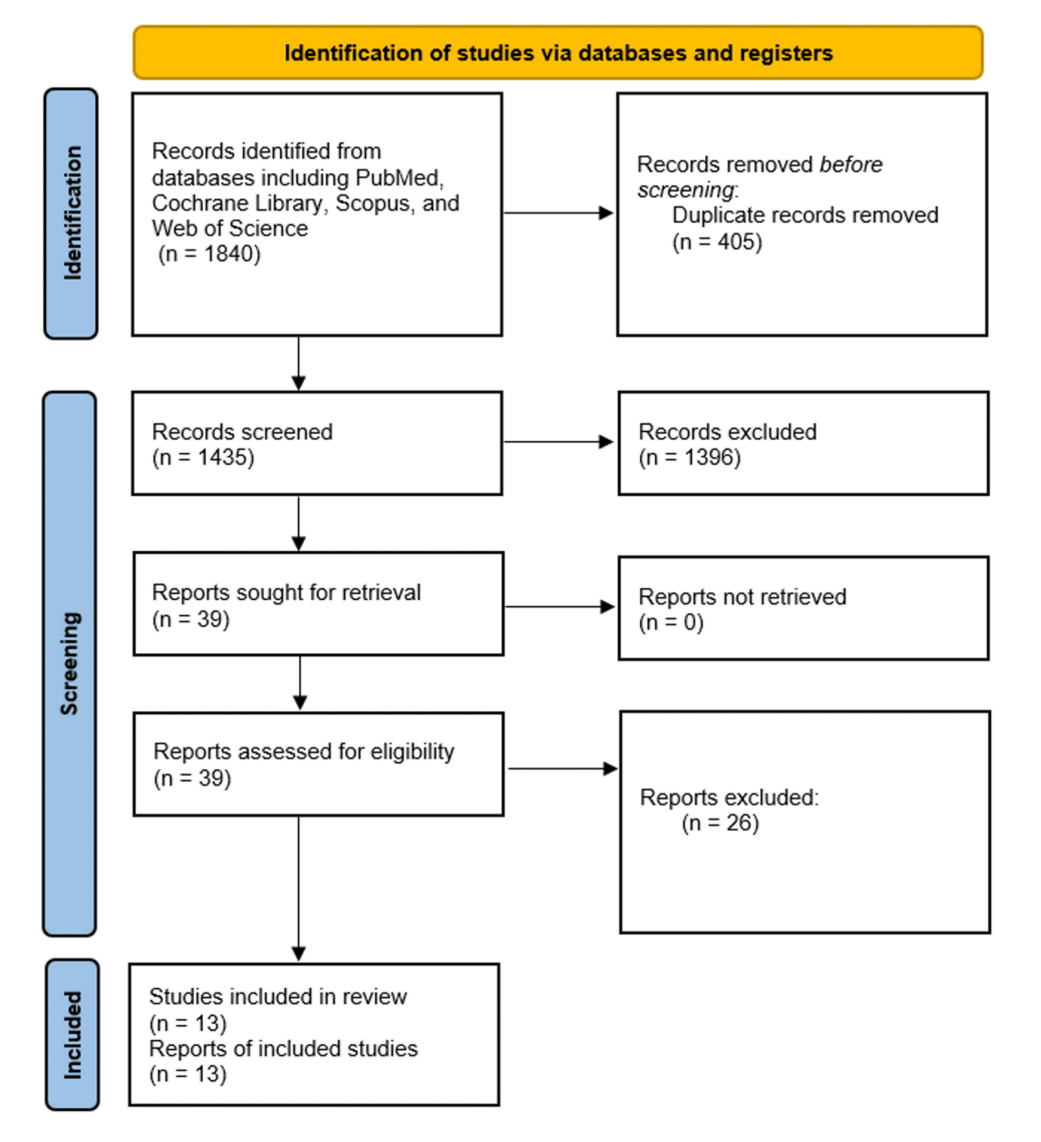
Flow diagram of the study selection process

The systematic search across various databases initially identified 1,840 articles. After the removal of duplicates, 1,435 unique articles underwent title and abstract screening. From these, 39 articles were selected for a detailed full-text review. All selected articles were retrieved; however, additional exclusions were made based on reasons such as failure to meet the inclusion criteria, insufficient data, poor study quality, or being review articles. Ultimately, 13 studies met the eligibility criteria and were included in the meta-analysis [12-24] (Figure 1).

Baseline Characteristics

The study encompassed studies with diverse characteristics, including various geographic regions, patient groups, and treatment approaches. The size of studies varied significantly, ranging from small trials with fewer than 20 participants per group to large-scale research involving over 500 subjects, such as the study by Shaikh et al. [20]. Patient ages spanned from approximately 64.9 years to over 80 years, primarily focusing on elderly individuals. While most studies reported similar gender distributions between treatment and control groups, some failed to provide this information. The majority of studies had a one-year follow-up period, with exceptions like Martínez-Mendez et al. [17], which extended to two years, and another study, which lasted only three months. Surgical interventions included open reduction using volar locking plate systems (VLPS), external fixation, and closed reduction with additional percutaneous pinning (K-wires), while conservative treatments consistently employed closed reduction and cast immobilization (Table 1).

**Table 1:** Baseline characteristics of the included studies VLPS: volar locking plate systems

Author	Year	Country	Sample size		Gender		Age		Follow-up time	Intervention	Control
			Intervention	Control	Intervention	Control	Intervention	Control			
Arora et al. [12]	2011	Austria	36	37	28	27	75.9 (65-88)	77.4 (65-89)	1 year	Open reduction with VLPS. After surgery, the wrist was immobilized in a below-the-elbow splint for pain reduction. Physiotherapy	Closed reduction and cast immobilization
Bartl et al. [13]	2014	Germany	86	87	77	76	75.3 (6.7)	74.4 (7.1)	1 year	Open reduction with VLPS and immobilization	Closed reduction and cast immobilization
Chan et al. [14]	2014	Singapore	40	35	34	30	71.5 (5.2)	75.8 (9.3)	1 year	Open reduction with VLPS and immobilization	Closed reduction and cast immobilization
Hassellund et al. [15]	2021	Norway	50	50	NR	NR	73.4 (65-91)	73.9 (65-89)	1 year	Open reduction with VLPS and immobilization	Closed reduction and cast immobilization
Lawson et al. [16]	2021	Australia and New Zealand	81	85	70	75	70.5 (7)	71.3 (7.6)	1 year	Open reduction with VLPS and immobilization	Closed reduction and cast immobilization
Martínez-Mendez et al. [17]	2017	Spain	50	47	39	37	67 (8)	70 (7)	2 years	Open reduction with VLPS and below-the-elbow plaster splint	Closed reduction and cast immobilization
Saving et al. [18]	2019	Sweden	68	78	56	55	80 (70-90)	78 (70-98)	1 year	Open reduction with VLPS and immobilization	Closed reduction and cast immobilization
Wong et al. [19]	2010	China	30	30	24	25	70 (66-76)	71 (65-76)	1 year	Closed reduction and supplementary percutaneous pinning (K-wires)	Closed reduction and cast immobilization
Shaikh et al. [20]	2023	Pakistan	534	529	326	330	64.90 (3.70)	65.16 (3.90)	1 year	Open reduction with VLPS and below-the-elbow plaster splint	Closed reduction and cast immobilization
Ahmed et al. [21]	2024	India	17	17	9	4	67.2±5.64	64.6±4.89	1 year	Open reduction with VLPS and below-the-elbow plaster splint	Closed reduction and cast immobilization
Haslhofer et al. [22]	2024	Austria	40	40	NR	NR	72.5 (65-83)	77.3 (67-87)	1 year	Open reduction with VLPS and below-the-elbow plaster splint	Closed reduction and cast immobilization
Tahir et al. [23]	2021	Pakistan	87	72	71	55	81±3	81±2	2 years	Anterior locking plate	Closed reduction and cast immobilization
Chung et al. [24]	2020	Canada, Singapore, and the United States	65	109	55	93	67 (6.2)	76 (10)	1 year	Open reduction with VLPS and immobilization	Closed reduction and cast immobilization

Wrist Function

**Figure 2:**
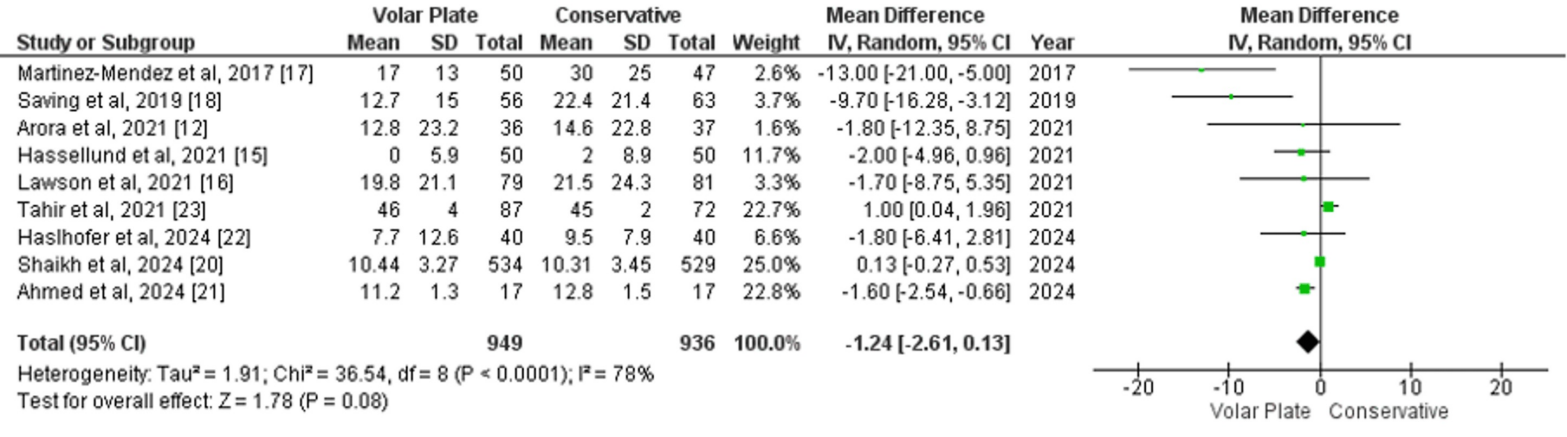
Forest plot of mean difference in wrist function at one-year follow-up (PRWE) between surgical and conservative treatments PRWE: Patient-Rated Wrist Evaluation

Wrist function at the one-year follow-up was assessed using the PRWE score across nine studies. The pooled analysis revealed an MD of -1.24 (95% CI -2.61 to 0.13; p=0.78), suggesting slightly better wrist function in the volar plate group compared to conservative treatment. However, the CI crossed zero, indicating that the difference was not statistically significant. Significant heterogeneity was observed (I²=78%) (Figure 2). A sensitivity analysis was performed by excluding studies contributing to high heterogeneity, specifically Martinez-Mendez et al. [17], Saving et al. [18], and Ahmed et al. [21]. This reduced heterogeneity to 17%, but the result remained statistically insignificant (MD=0.23; p=0.48), indicating no meaningful difference in wrist function between the two groups (Appendix 1).

Upper Limb Function

**Figure 3:**
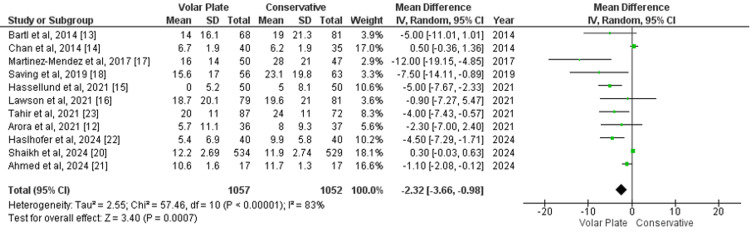
Forest plot of mean difference in upper limb function at one-year follow-up (DASH) between surgical and conservative treatments DASH: Disabilities of the Arm, Shoulder, and Hand

Upper limb function at the one-year follow-up, as measured by the DASH score, was reported in 11 studies. The pooled analysis showed an MD of -2.32 (95% CI -3.66 to -0.98; p=0.0007), favoring the volar plate group over conservative treatment. However, significant heterogeneity was observed (I²=83%) (Figure 3). A sensitivity analysis was conducted by excluding studies contributing to high heterogeneity, specifically Chan et al. [14], Martinez-Mendez et al. [17], Hassellund et al. [15], and Shaikh et al. [20]. Following these exclusions, heterogeneity decreased to 46%, and the MD further favored the volar plate group at -3.04 (95% CI -4.87 to -1.21; p=0.001), indicating a statistically significant improvement in upper limb function with volar plate treatment (Appendix 2).

Grip Strength

**Figure 4:**
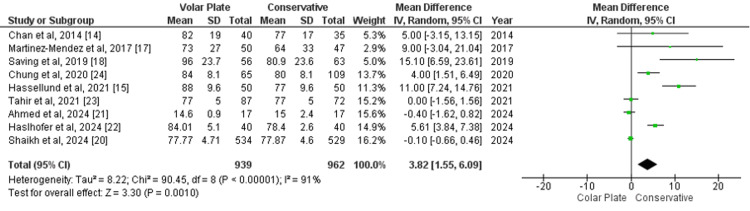
Forest plot of mean difference in grip strength at one-year follow-up between surgical and conservative treatments

Grip strength at the one-year follow-up, measured using a dynamometer, was assessed in nine studies. The pooled analysis revealed an MD of 3.82 (95% CI 1.55 to 6.09; p=0.0010), indicating a statistically significant advantage for the volar plate group compared to conservative treatment. However, substantial heterogeneity was noted (I²=91%) (Figure 4). To address the high heterogeneity, a sensitivity analysis was performed by excluding Saving et al. [18], Chung et al. [24], Hassellund et al. [15], and Haslhofer et al. [22]. After these exclusions, the results were no longer statistically significant (MD=-0.10; 95% CI -0.59 to 0.38; p=0.67), and heterogeneity was completely eliminated (I²=0%) (Appendix 3). This suggests that the initial significant result may have been influenced by the variability across studies.

Range of Wrist Flexion

**Figure 5:**
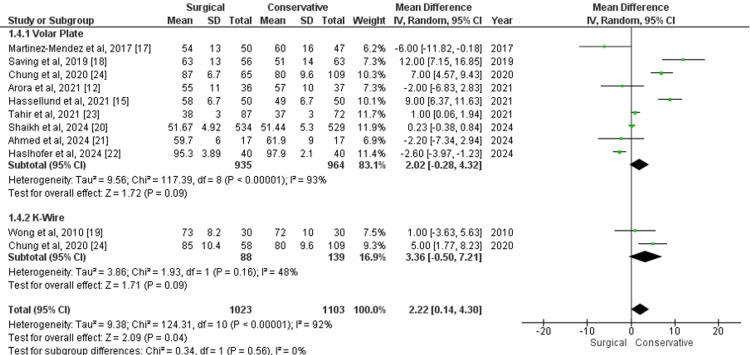
Forest plot of mean difference in range of wrist flexion at one-year follow-up between surgical and conservative treatments

The range of wrist flexion at the one-year follow-up, measured using a goniometer, was reported in 11 studies, including nine comparing volar plate with conservative treatment and two comparing K-wire with conservative treatment. The overall pooled analysis across all studies showed an MD of 2.22 (95% CI 0.14 to 4.30; p=0.04), favoring surgical treatment over conservative treatment, though with substantial heterogeneity (I²=92%) (Figure 5). Subgroup analysis revealed that for volar plate versus conservative treatment, the MD was 2.02 (95% CI -0.28 to 4.32; p=0.93), with high heterogeneity (I²=93%), whereas for K-wire versus conservative treatment, the MD was 3.36 (95% CI -0.50 to 7.21; p=0.09), with moderate heterogeneity (I²=48%). These findings suggest that while surgical treatments may offer a slight advantage in wrist flexion, the results are influenced by high variability and are not consistently statistically significant.

Range of Wrist Extension

**Figure 6:**
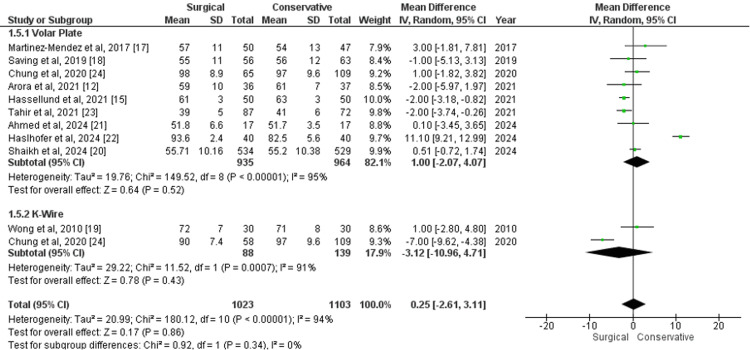
Forest plot of mean difference in range of wrist extension at one-year follow-up between surgical and conservative treatments

The range of wrist extension at the one-year follow-up, measured using a goniometer, was evaluated in studies comparing surgical and conservative treatments. For volar plate versus conservative treatment, the MD was 1.00 (95% CI -2.07 to 4.07; p=0.52), with substantial heterogeneity (I²=95%). In studies comparing K-wire versus conservative treatment, the MD was -3.12 (95% CI -10.96 to 4.71; p=0.43), also with high heterogeneity (I²=91%). The overall analysis across all surgical approaches versus conservative treatment showed an MD of 0.25 (95% CI -2.61 to 3.11; p=0.86), with significant heterogeneity (I²=94%) (Figure 6). These results indicate no statistically significant difference in wrist extension between surgical and conservative treatments at the one-year follow-up.

Pain Intensity

**Figure 7:**
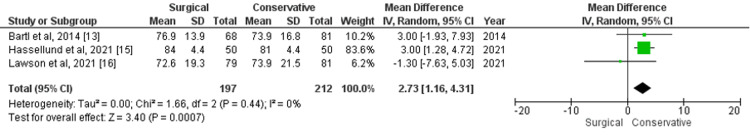
Forest plot of mean difference in pain intensity at one-year follow-up between surgical and conservative treatments

Pain intensity at the one-year follow-up was reported in three studies comparing surgical and conservative treatments. The pooled analysis revealed an MD of 2.73 (95% CI 1.16 to 4.31; p=0.0007), indicating significantly higher pain intensity in the surgical group. No heterogeneity was observed (I²=0%), suggesting consistency across the included studies (Figure 7).

Range of Wrist Pronation

**Figure 8:**
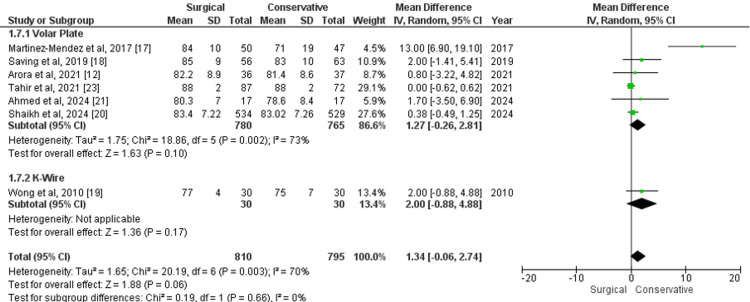
Forest plot of mean difference in range of wrist pronation at one-year follow-up between surgical and conservative treatments

The range of wrist pronation at the one-year follow-up, measured using a goniometer, was evaluated in studies comparing surgical and conservative treatments. For volar plate versus conservative treatment, the MD was 1.27 (95% CI -0.26 to 2.81; p=0.10), with moderate heterogeneity (I²=73%). In the comparison between K-wire and conservative treatment, the MD was 2.00 (95% CI -0.88 to 4.88; p=0.17), with data from only one study. The overall analysis across all surgical treatments versus conservative care showed an MD of 1.34 (95% CI -0.06 to 2.74; p=0.06), with moderate heterogeneity (I²=70%) (Figure 8). These results suggest a slight advantage for surgical treatment in wrist pronation, though the differences were not statistically significant across the outcomes.

Range of Wrist Supination

**Figure 9:**
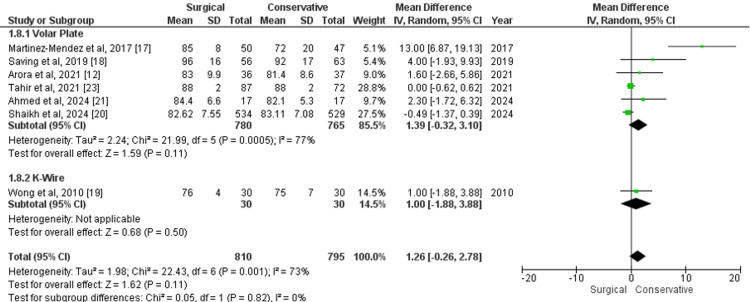
Forest plot of mean difference in range of wrist supination at one-year follow-up between surgical and conservative treatments

The range of wrist supination at the one-year follow-up, measured using a goniometer, was assessed in studies comparing surgical and conservative treatments. For volar plate versus conservative treatment, the MD was 1.39 (95% CI -0.32 to 3.10; p=0.11), with moderate heterogeneity (I²=77%). In the comparison between K-wire and conservative treatment, the MD was 1.00 (95% CI -1.88 to 3.88; p=0.50), based on data from only one study. The overall analysis across all surgical treatments versus conservative care showed an MD of 1.26 (95% CI -0.26 to 2.78; p=0.11), with moderate heterogeneity (I²=73%) (Figure 9). These results suggest no statistically significant difference in wrist supination between surgical and conservative treatments at the one-year follow-up, though there is a slight favoring trend towards surgical approaches.

Risk of Bias

**Figure 10:**
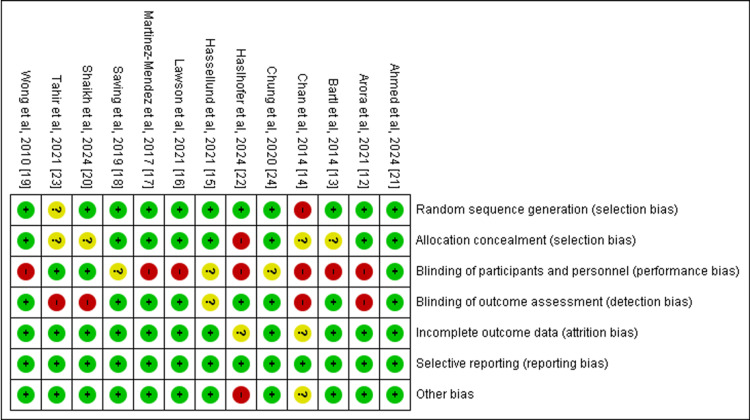
Risk of bias summary

**Figure 11:**
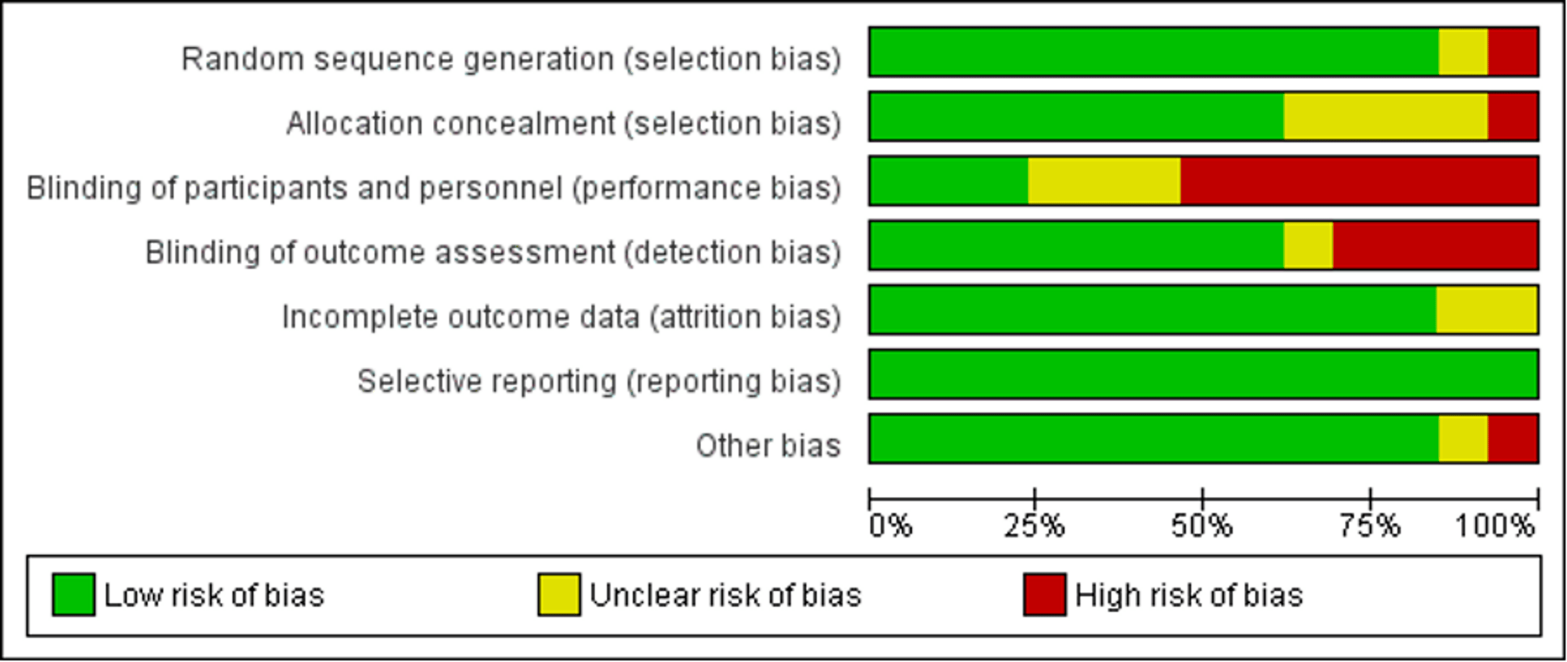
Risk of bias graph

The risk of bias across the included studies varied. Ahmed et al. [21] were assessed as having a low risk across all domains. Several studies had a high risk of bias in specific domains, including Arora et al. [12] (performance and detection bias), Chan et al. [14] (selection, performance, and detection bias), Haslhofer et al. [22] (selection, performance, and other bias), Lawson et al. [16] (performance bias), Martinez-Mendez et al. [17] (performance bias), and Wong et al. [19] (performance bias). Other studies exhibited an unclear risk in certain domains, such as Bartl et al. [13] (selection bias and performance bias), Chung et al. [24] (performance bias), Hassellund et al. [15] (performance and detection bias), Saving et al. [18] (performance bias), Shaikh et al. [20] (selection bias and detection bias), and Tahir et al. [23] (selection and detection bias). These findings suggest varying levels of potential bias across the studies, with performance and detection bias being the most common concerns (Figure 10 and Figure 11).

Publication Bias

**Figure 12:**
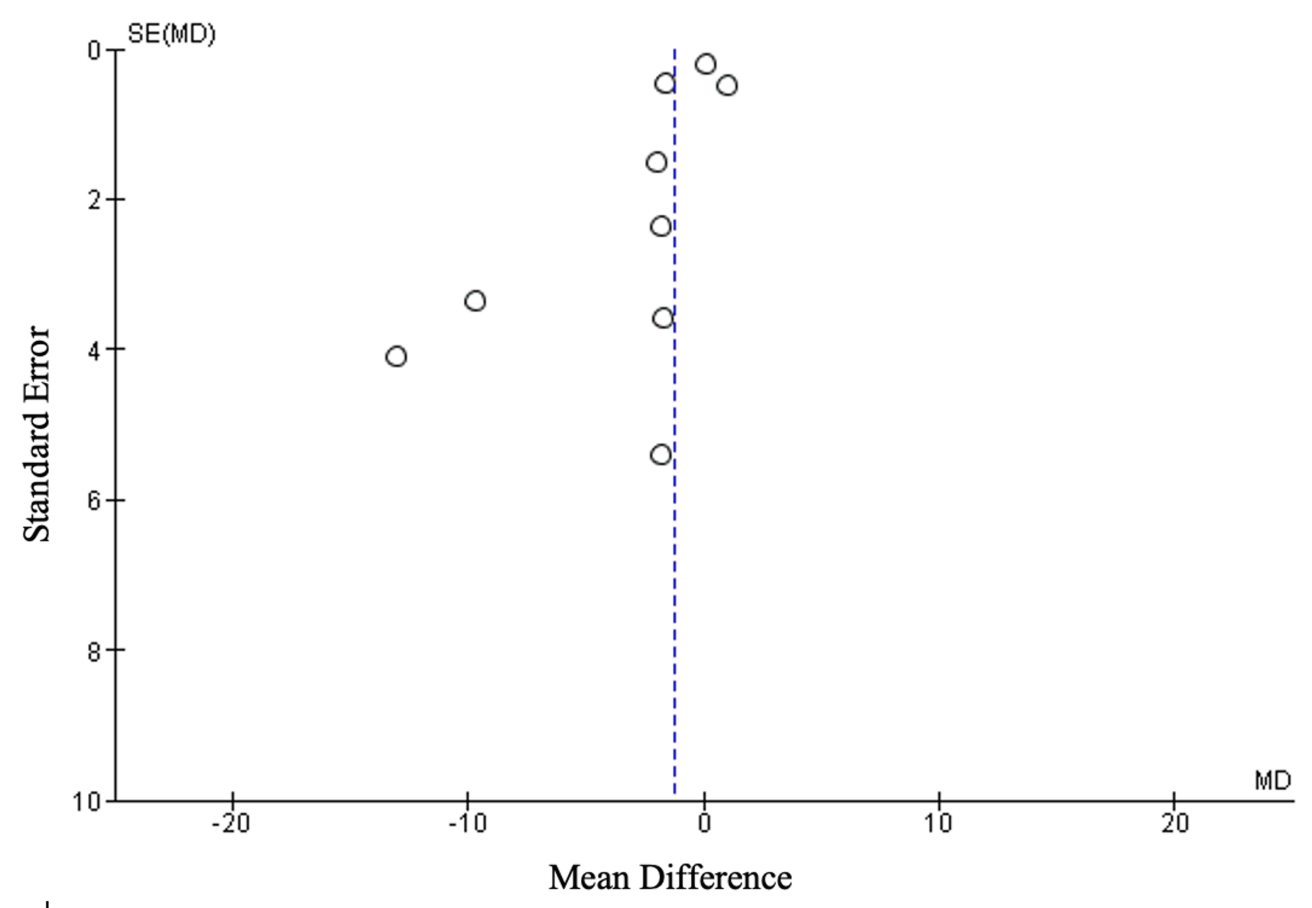
Funnel plot assessing publication bias for primary outcome

Publication bias was assessed for the primary outcome of wrist function. The funnel plot for this outcome displayed clear asymmetry, suggesting the presence of publication bias. The asymmetry indicates that studies with favorable results may be more likely to be published, while studies with negative or inconclusive results may remain unpublished. This could potentially skew the overall findings, as smaller studies or those with less significant outcomes might be underrepresented in the analysis (Figure 12).

Discussion

DRFs rank among the most prevalent injuries in the elderly population, typically resulting from low-energy falls due to decreased bone mass and age-related frailty [25,26]. The management of these fractures poses a significant clinical challenge, with both surgical and non-surgical approaches widely employed but often debated. Operative techniques, such as volar plate fixation and K-wire fixation, aim to restore anatomical alignment and enable early mobilization [27-29]. In contrast, conservative management using casts or splints is less invasive and more economical, relying on the body's innate healing capacity [29-31]. While surgical interventions are frequently believed to yield superior functional outcomes, they also entail greater risks and costs. This comprehensive review and meta-analysis are crucial as they seek to resolve this controversy by synthesizing evidence from RCTs to assess functional results, pain scores, and other key parameters between the two approaches. By focusing on older adults (65 years and above), this study addresses a particularly vulnerable demographic that is often underrepresented in broader orthopedic research. The objective of this analysis is to provide insights into the most effective treatment strategy for this population while also highlighting gaps in existing research, proposing directions for future investigations, and guiding clinical decision-making.

This analysis examined 13 RCTs involving a wide range of patients and methodological approaches. The results revealed complex findings. Surgical procedures showed slight enhancements in upper extremity function, hand grip strength, and wrist flexion range, especially when evaluated using validated instruments such as the DASH score and dynamometers. Nevertheless, the disparities in wrist functionality, as assessed by the PRWE, were not statistically meaningful between surgical and conservative management. Pain levels at the one-year follow-up were marginally higher in surgically treated groups, although this trend was consistent across studies.

The minor advantage of surgical interventions in certain functional aspects can be attributed to the mechanical benefits of internal fixation. Surgical stabilization corrects the anatomical positioning of fractured bone segments, facilitating early movement and minimizing the likelihood of joint rigidity [32,33]. This is crucial for complex or joint-involving fractures, where non-surgical approaches may not achieve proper alignment. Moreover, robust fixation methods like volar plating provide wrist stability during the initial healing stages, enabling patients to recover strength and mobility more quickly [34-36]. In contrast, conservative treatments depend on immobilization, which promotes natural bone healing but often leads to complications such as joint stiffness, muscle wasting, and delayed functional recovery [37-39]. These effects are more pronounced in older patients, who typically experience slower healing due to age-related decreases in bone and tissue regeneration abilities. Imperfect fracture alignment in conservatively managed patients may also result in lasting discomfort and reduced functionality. Pain outcomes further highlight the fundamental differences between these treatment strategies. While surgeries offer anatomical benefits, they involve soft tissue manipulation and hardware insertion, potentially causing extended postoperative pain and inflammation. Conversely, conservative approaches avoid these invasive procedures but may result in malunion or persistent deformities, possibly leading to long-term pain or arthritis.

Multiple meta-analyses have explored the outcomes of surgical versus non-surgical management of DRFs, highlighting varied perspectives on the efficacy of these approaches. A study conducted a detailed comparison of internal and external fixation methods, concluding that internal fixation was superior to external fixation in terms of postoperative complications, clinical outcomes, and radiological results [40]. Their findings emphasized the advantages of open internal fixation in achieving better anatomical alignment and functional recovery. Similarly, another study assessed the efficacy of external versus internal fixation for DRFs [41], reporting that open reduction and internal fixation (ORIF) was associated with improved follow-up outcomes, supporting its use in certain patient populations. Despite these findings, not all research aligns with the notion that surgical treatment yields superior clinical outcomes. Another study concluded that both management strategies produced comparable results [42]. Their analysis suggested that while surgical intervention might offer benefits in some aspects, conservative treatment remains an equally effective approach in many cases, challenging the presumption of surgical superiority for DRFs.

In contrast to this study's results, a previous systematic review indicated no significant clinical or statistical advantages of surgical approaches over closed reduction and cast immobilization for elderly patients with DRFs [43]. However, the present meta-analysis shows a slight benefit favoring surgical treatment. Notably, a recent network meta-analysis supports earlier findings, advocating for conservative management as the preferred option for patients above 60 years old, citing insufficient evidence of reduced complications or surgical revision rates with operative methods [44]. While these findings do not negate the importance of surgical intervention, they emphasize the need to consider the specific requirements of older adults, including their reduced functional demands and the primary goal of maintaining quality of life. For this age group, conservative treatment may be adequate to achieve satisfactory outcomes without the risks linked to surgical procedures, especially given increasing life expectancy. Therefore, decisions regarding surgery for this demographic should be made cautiously, weighing potential benefits against the inherent risks of operative intervention.

While this meta-analysis provides a thorough evaluation of treatments for DRFs, it has several limitations. The considerable variability across outcomes underscores the range of study methods, patient populations, and treatment strategies. Although sensitivity analyses were employed to address this issue, some heterogeneity persisted, impacting the reliability of pooled results. By limiting the analysis to RCTs, the study improved methodological rigor but may have excluded valuable information from observational and real-world studies. The focus on one-year outcomes restricts our knowledge of long-term complications and functional recovery. Moreover, the risk of bias in the included studies, especially regarding performance and detection, raises concerns about the robustness of individual study findings. The possibility of publication bias, as suggested by asymmetrical funnel plots, indicates that studies with less favorable outcomes might be underrepresented, potentially distorting the overall conclusions. Lastly, the concentration of studies from high-income countries limits the generalizability of findings to low- and middle-income settings where surgical resources may be limited.

Despite its limitations, this research possesses notable strengths. It provides a focused analysis of elderly patients, addressing a demographic often underrepresented in orthopedic research. The rigorous methodology, including a comprehensive literature review and adherence to Cochrane guidelines for data gathering and analysis, ensures the inclusion of high-quality evidence. By synthesizing results from multiple studies, this meta-analysis delivers a holistic view of treatment outcomes, aiding healthcare professionals in making evidence-based decisions. Future studies should prioritize long-term follow-up investigations to evaluate lasting functional outcomes and complications. Standardizing outcome measures across studies would enhance comparability and facilitate more reliable meta-analyses. Subgroup analyses based on fracture complexity, patient comorbidities, and rehabilitation protocols are essential for tailoring treatment strategies. Economic evaluations comparing the cost-effectiveness of surgical and conservative approaches would provide valuable insights for policymakers, while an increased emphasis on patient-reported outcomes would ensure treatment decisions align with patient preferences. Additionally, exploring emerging technologies, such as personalized implants or advanced physiotherapy techniques, could revolutionize fracture management.

## Conclusions

This comprehensive review and statistical analysis offer an in-depth evaluation of surgical versus conservative approaches for treating DRFs in older adults, revealing subtle distinctions in functional results, pain levels, and recovery patterns. Although surgical procedures may yield modest improvements in upper extremity function and grip strength, these potential benefits must be carefully considered against increased pain, higher expenses, and surgical complications. Non-surgical treatments remain a feasible alternative, especially for individuals seeking less invasive options or those with medical contraindications to surgery. The selection of treatment should be tailored to each patient's specific circumstances, including fracture severity, general health status, and personal preferences. This investigation emphasizes the necessity of an individualized strategy for fracture management, backed by robust scientific evidence. Addressing existing knowledge gaps through extended, standardized, and patient-focused research will be crucial for enhancing orthopedic care and improving outcomes in elderly patients with DRFs.

## Figures and Tables

**Figure 13:**
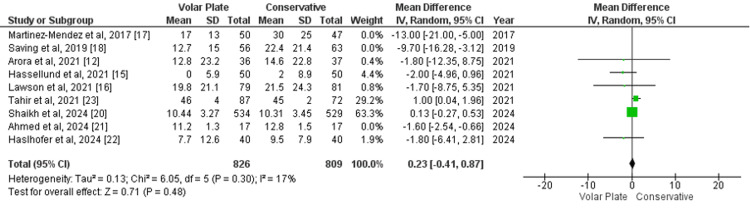
Sensitivity analysis of Figure 2

**Figure 14:**
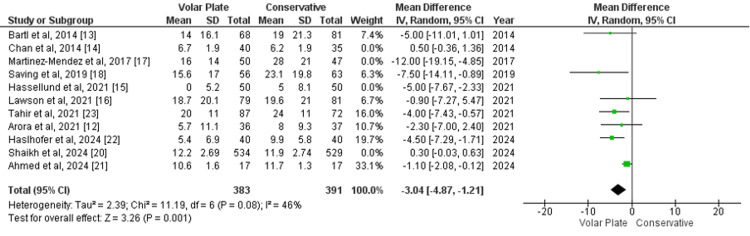
Sensitivity analysis of Figure 3

**Figure 15:**
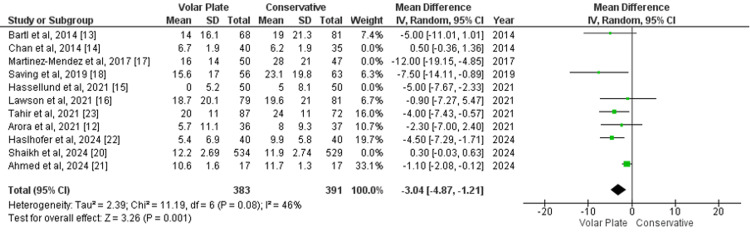
Sensitivity analysis of Figure 4

## Appendices

Appendix 1

Appendix 2

Appendix 3
